# Dimensionality and psychometric analysis of DLQI in a Brazilian population

**DOI:** 10.1186/s12955-020-01523-9

**Published:** 2020-08-05

**Authors:** Marilia F. S. Jorge, Ticiane D. Sousa, Camila F. Pollo, Bianca S. R. Paiva, Mayra Ianhez, Juliana C. Boza, Silmara Meneguin, Juliano V. Schmitt, Daniel Holthausen Nunes, Joel C. Lastoria, Hélio A. Miot

**Affiliations:** 1 Departamento de Dermatologia da Faculdade de Medicina da Unesp, Unesp Medical School, Campus Universitário de Rubião Jr, Botucatu, SP 18618-000 Brazil; 2 Departamento de Enfermagem, Unesp Medical School, Botucatu, SP, Brazil, Campus Universitário de Rubião Jr, Botucatu, SP 18618-000 Brazil; 3 Health-Related Quality of Life Research Group (GPQual), Learning and Research Institute - Barretos Cancer Hospital, Barretos, SP 14784-40 Brazil; 4 Departamento de Dermatologia, Universidade Federal de Goiás (UFGO), Av. Esperança, s/n - Chácaras de Recreio Samambaia, Goiânia, GO 74690-900 Brazil; 5 Departamento de Dermatologia, Universidade Federal do Rio Grande do Sul (UFRGS), Av. Paulo Gama, 110 – Farroupilha, Porto Alegre, RS 90040-060 Brazil; 6 Disciplina de Dermatologia, Universidade Federal de Santa Catarina (UFSC), R. Eng. Agronômico Andrei Cristian Ferreira, s/n – Trindade, Florianópolis, SC 88040-900 Brazil

**Keywords:** Dermatology, Psychometrics, Questionnaires, Health-related quality of life, Validity, Factor analysis

## Abstract

**Background:**

The Dermatology Life Quality Index (DLQI) is the most commonly used instrument for clinical evaluation of the impact on health-related quality of life (HRQOL) in dermatological research protocols. The DLQI’s classical psychometric properties have been considered adequate in validation studies from several countries. However, the structure of the DLQI is a matter of discussion, especially concerning the dimensionality and informative properties of its questions according to the item response theory (IRT).

**Methods:**

Pooled data from studies in Brazil that utilized the DLQI to assess HRQOL in 14 dermatoses were reanalyzed. Classical psychometrical analysis, dimensionality assessment through parallel analysis and IRT (Samejima’s ordinal model) analysis were performed.

**Results:**

The sample consisted of 1286 patients with a mean age of 47 years (*SD* = 16), and the proportion of women was 59% (765). The DLQI scores ranged from 0 to 29, with a median (p_25_–p_75_) of 5 (2–11). All items indicated significant correlations with the total DLQI score (rho > 0.54). The Cronbach’s alpha result was 0.90 (CI 95% 0.89–0.91). Parallel analysis indicated a unidimensional factor structure. According to IRT analysis, items q6 (sports) and q7 (work/study) exhibited insufficient fit to the model (*p* < 0.01), while the items that indicated the best discrimination and information functions were q2 (embarrassment), q3 (shopping/gardening), q4 (clothing) and q5 (social/leisure). The ordination of the scores was confirmed for all items. Most items revealed non-uniform behavior according to sex, age and type of disease.

**Conclusions:**

The DLQI exhibits adequate psychometric reliability and a unidimensional structure for assessing HRQOL in Brazilian dermatological patients. The DLQI’s performance varies in the assessment of HRQOL in heterogeneous samples.

## Introduction

Most dermatologic diseases are not life-threatening or extremely symptomatic; however, they affect an individual’s social, affective and emotional functioning. Thus, patient-oriented outcomes are important in measuring disease burden and substantiating therapeutic efficacy and decisions on treatment. The appropriateness of the instruments assessing health-related quality of life (HRQOL) rest on their validity, consistency, reliability, dimensionality and invariance through subgroups [1]. The Dermatology Life Quality Index (DLQI) is the most commonly used instrument for the HRQOL evaluation and follow-up of patients with dermatological diseases under research protocols; it is also part of the decision-making algorithm for several treatment guidelines. Nevertheless, there are concerns regarding its performance, which we examine [2–4].

The DLQI is a short HRQOL questionnaire on general dermatological diseases. It was published in 1994 by Finlay and Khan [5], and it has been translated into more than 90 languages and applied to research on more than 40 dermatoses. It is a practical and straightforward instrument that has performed well in several studies [6–10]. In 2004, Martins et al. validated the DLQI for the Portuguese language in Brazil (DLQI-BRA) [11, 12].

The DLQI was developed from interviews with 120 patients with different dermatological diseases, in which the patients highlighted aspects that affected their daily lives. From this qualitative approach, the 10 most important aspects were selected. To validate the adequacy of the psychometric properties, 200 dermatological patients and 100 controls were assessed [5]. Theoretically, the DLQI is a scale developed as a unidimensional reflexive instrument to assess dermatologic diseases’ effects on HRQOL.

The final version of the DLQI consists of 10 items arranged in six categories: symptoms and feelings (questions 1 and 2), daily activity (3 and 4), leisure (5 and 6), work or study (7), interpersonal relationships (8 and 9) and treatment (10). The questions evaluate an individual’s perception of the disease over the past week. The possible answers for each item are “very much,” “a lot,” “a little,” “not at all” and “not relevant,” with a respective ordinal grade of 0 to 3. Item 7 is divided into two stages: the first stage questions whether skin disease prevents the individual from working; if the answer is no, the next step asks how much the disease interferes with his or her work [13]. The total score can vary from 0 (no impact on HRQOL) to 30 (maximum impact on HRQOL) [5, 10]. Conventionally, DLQI scores are interpreted from the sum of the indices of the 10 items evaluated, such as “no impairment of HRQOL” (0–1), “mild impairment” (2–5), “moderate” (6–10), “severe” (11–20) or “very severe impairment” (21–30) [14].

The psychometric properties regarding classical test theory have been considered appropriate for evaluating HRQOL in patients with dermatological diseases in studies in several countries. However, the structure of the DLQI is currently a matter of discussion, especially regarding the informative properties of its items according to the item response theory (IRT) [15–17].

Recent studies have not recognized the unidimensionality of the DLQI. Moreover, differential item functioning (DIF) analysis reveals that the same item presents different behavior according to age, sex and the type of dermatological disease [13, 18–20].

The content validity of the DLQI has been questioned due to the instrument’s insufficient evaluation of emotional and psychological aspects, which are fundamental in dermatology, especially in asymptomatic but stigmatizing diseases, such as vitiligo, congenital nevus, melasma and alopecia [1, 21–24]. Furthermore, the plurality of dermatological diseases affects different dimensions of HRQOL. The DLQI’s unidimensional proposal may not capture all the nuances in different dermatoses.

This study aims to investigate the DLQI’s internal consistency, dimensionality, discrimination and performance in a subgroup analysis of a Brazilian population sample.

## Methods

We performed a reanalysis of pooled data from cross-sectional studies in Brazil that utilized the DLQI-BRA to assess the HRQOL impact of 14 dermatoses on 1286 patients [25–33]. Furthermore, sex, age, educational status and physical and psychological dimensions of the skin disorders were evaluated. The authors of these original studies provided permission to reanalyze the data, and all these projects were approved by their institutional review boards.

The dermatoses were classified (by author consensus) according to characteristic physical symptoms and psychological or social domains (Table 1) to test the performance of the DLQI items and scores in different types of skin disorders. This classification was chosen based on the dimensions of another validated HRQOL multidimensional scale (Skindex-17) [34].

**Table 1:** Classification of dermatoses regarding physical/symptomatic and psychological/social dimensions

Skin disorder	Physical/Symptomatic	Psychological/Social
Basal cell carcinoma		x
Bullous disorders	x	x
Female alopecia		x
Genital warts		x
Hidradenitis suppurativa	x	x
Leprosy	x	x
Melasma		x
Onychocriptosis	x	
Photoaging		x
Psoriasis	x	x
Rosacea		x
Uremic pruritus	x	
Urticaria	x	
Vitiligo		x

As the score of the DLQI results from the sum of its 10 items, the assurance of unidimensionality is crucial. The DLQI dimensionality was assessed by Horn’s parallel analysis method, using a random matrix (sphericity calculated after a Monte Carlo simulation method with 99% reliability). Additionally, the unidimensional congruence (UniCo), explained common variance (ECV) and mean of item residual absolute loadings (MIREAL) were assessed, and the Hull method was performed [35–40]. The internal consistency of the DLQI was estimated by Cronbach’s alpha and its 95% confidence interval (CI 95%). Furthermore, McDonald’s ordinal omega and the greatest lower bound to reliability were assessed [41–43]. Inter-item and item-total correlations were measured by polychoric correlations and Spearman’s rho coefficients [44–46].

The normality of the data distribution was assessed by the Kolmogorov-Smirnov (Lilliefors) test. Quantitative variables are expressed as means (standard deviation) or medians and quartiles (p_25_-p_75_) [47].

To analyze the informativity of each item, the DLQI was evaluated according to the IRT through a graded response (Samejima’s) model [48]. The adjustment of the model was assessed by the Akaike information criterion (AIC), the Bayesian information criterion (BIC), the comparative fit index (CFI), the root mean square error of approximation (RMSEA) and X^2^. The coefficients for each item were then extracted.

Another important issue to assess is related to invariance in the measurement of people with equivalent abilities. The item invariability was assessed through DIF analysis by ordinal logistic regression for each item according to sex, age group (< 30, 30–60 and > 60 years old) and the characteristics of the disease [13, 49]. The total DLQI score was tested regarding these covariates by a generalized linear model (gamma regression).

The sample was a result of pooled data from 14 cross-sectional studies in Brazil that utilized the DLQI-BRA to assess HRQOL [25–33]. The sample size (*n* = 1286) was assumed to be sufficient in the IRT parameter estimation, dimensionality assessment and DIF and generalized linear model analyses, adjusted for up to eight dummy variables [50–52]. All data was related to completely filled questionnaires; there was no available information regarding the number of distributed questionnaires or the percentage of incomplete questionnaires in each original study.

Data were analyzed with IBM SPSS 25.0; Factor [53] and R (mirt package) [54]. Significance was set as two-tailed *p* < 0.05.

## Results

**Fig. 1:**
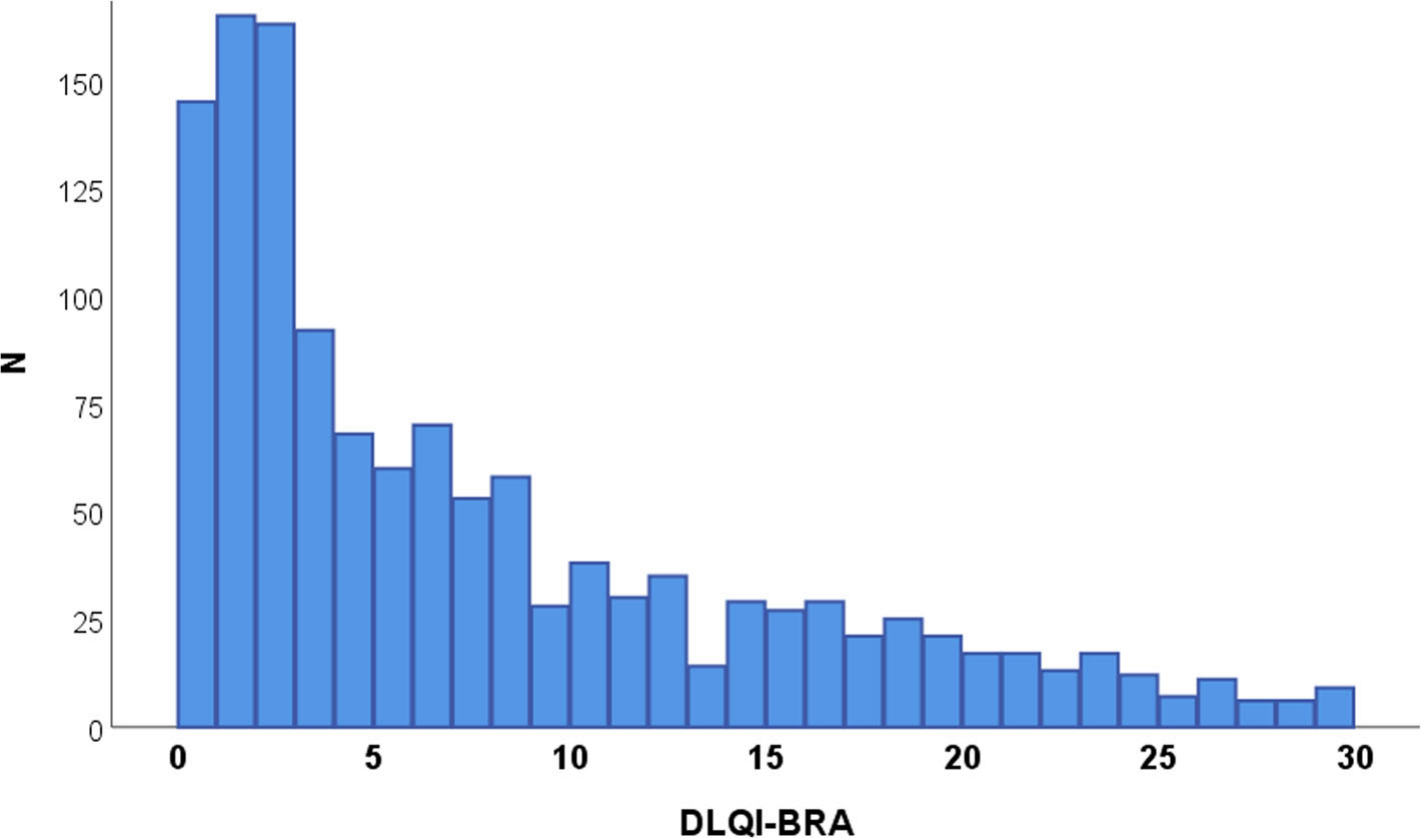
Histogram of DLQI-BRA scores (*n* = 1286)

The main clinical and demographic data from the sample are displayed in Table 2. The DLQI scores ranged from 0 to 29, with a median of 5 (p_25_ = 2; p_75_ = 11) (Fig. 1). According to the predefined categories of HRQOL impairment, 310 (24%) participants were classified as having no impairment, 383 (30%) were classified as having a mild impairment, 247 (19%) as having a moderate impairment, 248 (19%) as having a severe impairment and 98 (8%) as having a very severe impairment.

**Table 2:** Main demographic and quality of life data (DLQI-BRA) according to each dermatosis (*n* = 1286)

Dermatosis	***n*** (%)	Female (%)	Age^**a**^	Education level (%)			DLQI-BRA^**b**^	
				Elementary	High school	College		Cronbach’s alpha (CI 95%)
Melasma	143 (11)	123 (86)	39 (8)	19 (13)	42 (29)	82 (57)	3 (1–6)	0.89 (0.85–0.91)
Urticaria	101 (8)	87 (86)	42 (15)	62 (61)	35 (35)	4 (4)	14 (10–17)	0.73 (0.64–0.80)
Photoaging	100 (8)	64 (64)	63 (7)	69 (69)	25 (25)	6 (6)	1 (0–4)	0.72 (0.62–0.79)
Genital Warts	89 (7)	0 (−)	39 (14)	39 (44)	33 (37)	17 (19)	2 (0–4)	0.73 (0.64–0.81)
Bullous	91 (7)	56 (62)	47 (15)	70 (77)	16 (18)	5 (6)	16 (9–20)	0.81 (0.74–0.86)
Onychocriptosis	67 (5)	41 (61)	38 (17)	13 (19)	44 (66)	10 (15)	8 (5–12)	0.77 (0.68–0.85)
Uremic pruritus	65 (5)	24 (37)	61 (13)	35 (54)	27 (42)	3 (5)	4 (2–7)	0.84 (0.78–0.89)
FPHL^c^	76 (6)	76 (100)	47 (14)	18 (24)	13 (17)	45 (59)	6 (5–10)	0.88 (0.84–0.92)
Vitiligo	96 (8)	66 (69)	46 (17)	33 (34)	37 (39)	26 (27)	3 (1–7)	0.87 (0.82–0.90)
Psoriasis	135 (11)	62 (46)	51 (14)	68 (50)	44 (33)	23 (17)	3 (1–8)	0.91 (0.90–0.93)
Leprosy	92 (7)	31 (34)	51 (14)	75 (82)	15 (16)	2 (2)	3 (2–7)	0.89 (0.85–0.92)
Rosacea	72 (6)	59 (82)	45 (12)	17 (24)	20 (28)	35 (49)	5 (2–8)	0.85 (0.79–0.90)
BCC^d^	70 (5)	35 (50)	63 (11)	54 (77)	14 (20)	2 (3)	1 (1–3)	0.79 (0.70–0.85)
Hidradenitis	89 (7)	43 (48)	31 (13)	17 (19)	49 (55)	23 (26)	19 (12–25)	0.91 (0.88–0.93)
**TOTAL**	1286 (100)	767 (60)	47 (16)	589 (46)	414 (32)	283 (22)	5 (2–11)	0.90 (0.89–0.91)

^a^Mean (*SD*)

^b^Median (p25-p75)

^c^Female-pattern hair loss

^d^Basal cell carcinoma

Inter-item correlations were indicated for all items (> 0.3), and correlations between items and DLQI scores were found (Table 3). Sexual difficulties (q9) indicated the lowest item-total correlation (rho = 0.54), while shopping/home activities and social/leisure (q3 and q5) revealed the highest correlations (rho = 0.82).

**Table 3:** Inter-item polychoric correlation analysis and item-total Spearman’s rho correlation (*n* = 1286)

	q1	q2	q3	q4	q5	q6	q7	q8	q9	q10
**q1**		0.55	0.64	0.53	0.59	0.35	0.45	0.36	0.38	0.51
**q2**			0.79	0.67	0.78	0.54	0.45	0.57	0.55	0.56
**q3**				0.74	0.87	0.63	0.61	0.62	0.61	0.65
**q4**					0.76	0.61	0.48	0.57	0.58	0.58
**q5**						0.71	0.58	0.66	0.62	0.67
**q6**							0.41	0.56	0.53	0.59
**q7**								0.47	0.49	0.52
**q8**									0.73	0.54
**q9**										0.61
**DLQI-BRA**	0.66	0.79	0.82	0.73	0.82	0.56	0.57	0.59	0.54	0.59

All correlations produced *p-values* < 0.01

**Fig. 2:**
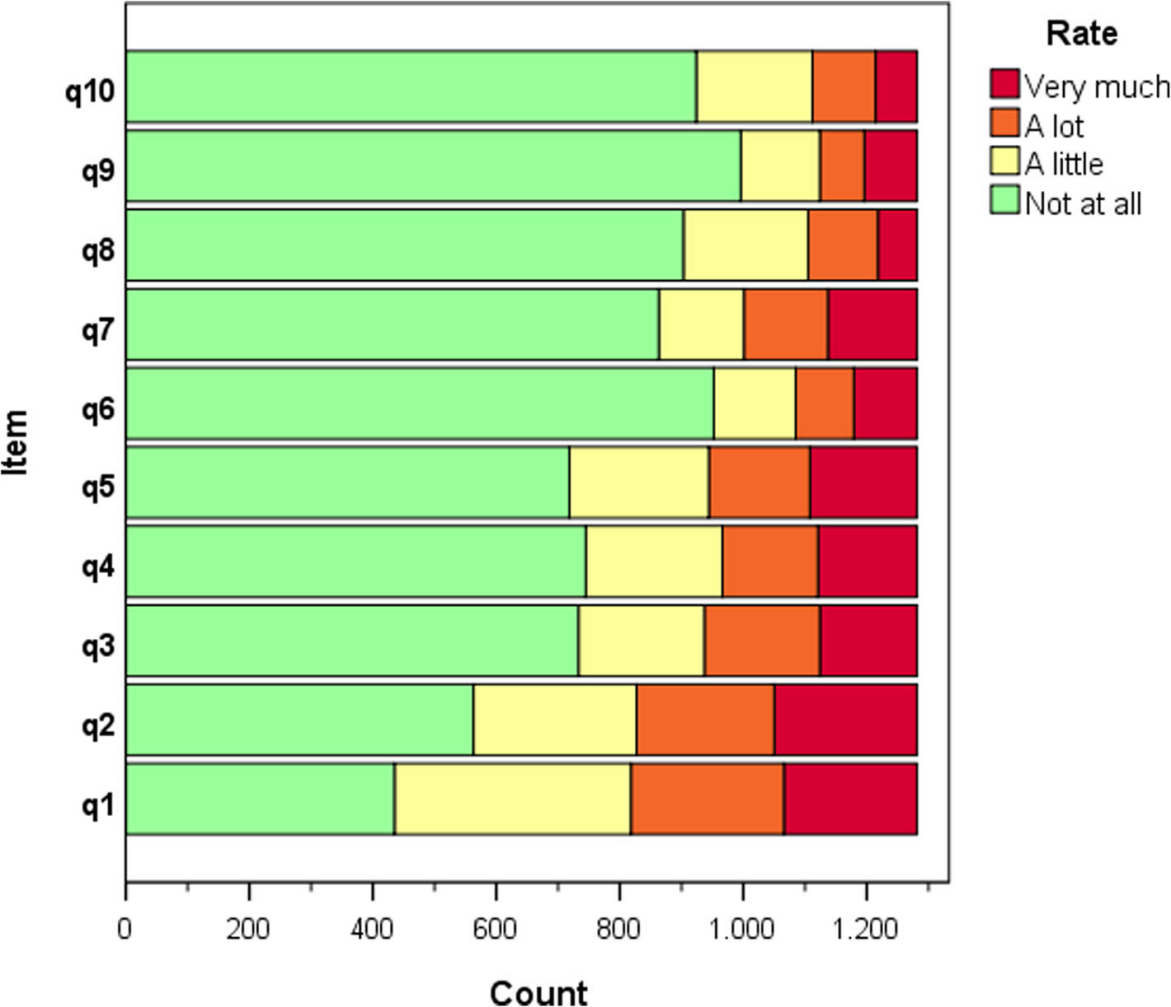
Distribution of item grades of the sample (*n* = 1286)

The distribution of item grades was asymmetrical for most items (Fig. 2), as was that of the total DLQI scores (Fig. 1). In more than 67% of cases, the rating of items q6 through q10 was “no” or “not at all.”

**Fig. 3:**
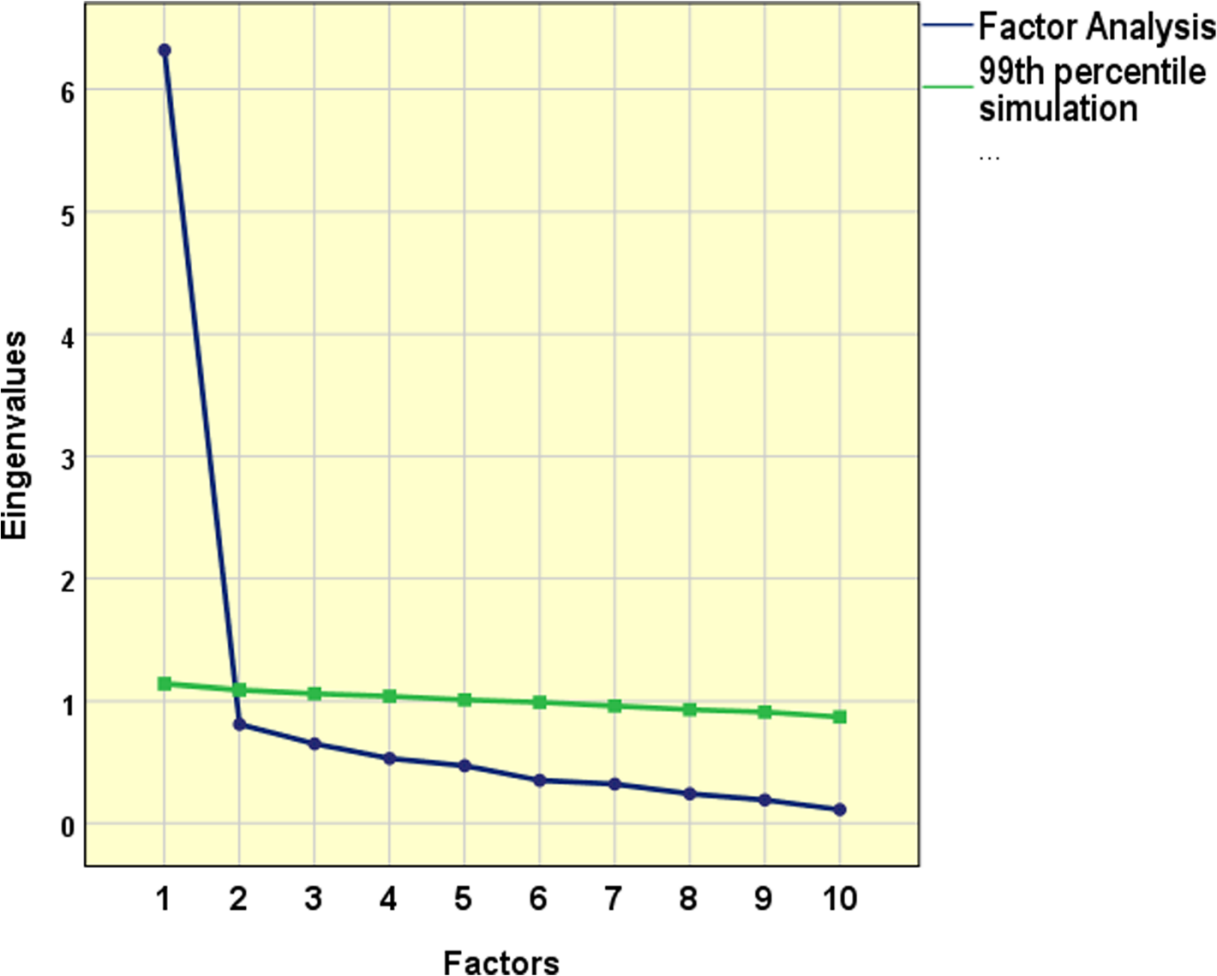
Diagram of the eigenvalues versus the number of factors, showing only one factor to the point of inflection and stabilization of the curve: analysis of scree plot; and before the crossing of the parallel analysis with a random spherical matrix: Horn’s parallel analysis (*n* = 1286; Kaiser-Meyer-Olkin test = 0.92; Bartlett’s statistic = 6219.9, df = 45; *p* < 0.01)

Horn’s parallel analysis and the scree plot pattern indicated unidimensionality for the DLQI (Fig. 3). The UniCo result was 0.99 (CI 95% 0.98–0.99), ECV was 0.91 (CI 95% 0.90–0.93) and MIREAL was 0.19 (CI 95% 0.16–0.21). Hull analysis also corroborated the unidimensional structure (data not shown). Moreover, when a second factor was extracted, the total explained variation increased from 63 to 71%.

The internal consistency of the DLQI (Cronbach’s alpha) was 0.90 (CI 95% 0.89–0.91), which varied from 0.72 to 0.91 according to the dermatoses (Table 2). If any item was excluded, Cronbach’s alpha for the total sample ranged from 0.87 to 0.89 (data not shown). Internal consistency was also greater than 0.8, as assessed by McDonald’s ordinal omega (0.90) and the greatest lower bound to reliability (0.91).

The DLQI was tested according to the ordinal IRT for four models: Samejima’s graded response model, the generalized partial credit model (GPCM), the graded ratings scale model (GRSM) and the Rasch rating scale model (RSM). Samejima’s graded response model produced the best adjustment (AIC = 22.157; CFI = 0.98; RMSEA = 0.05; X^2^ = 318.9; *p* = 0.22).

The coefficients from the items and the item-fit parameters are listed in Table 4. Items q6 and q7 exhibited unsatisfactory fit to the model (*p* < 0.01). All items demonstrated good discrimination (a > 0.8), and the items that disclosed the best discrimination (a > 2) and information functions were q2, q3, q4 and q5. The difficulty of the items followed a suitable ordination according to Samejima’s graded response model; however, the RSM and GRSM models revealed irregularities in the ordination of the response categories (data not shown). Items q1 and q2 (symptoms and feelings) identified subjects with lower levels of HRQOL impact (b1 < − 0.2), though item q9 (sexual life) identified only higher levels of HRQOL impact (b1 > 1.0).

**Table 4:** IRT coefficients of each item extracted from the graded response model (Samejima) and fit-adjusted parameters (chi-square)

Item	a	b1	b2	b3	chi-square	***p***
q1	1.40	−0.69	0.48	1.48	44.69	0.04
q2	2.55	−0.21	0.43	1.10	17.97	0.96
q3	4.22	0.18	0.44	1.27	15.49	0.99
q4	2.36	0.22	0.83	1.43	10.93	0.99
q5	4.74	0.14	0.68	1.20	13.62	0.99
q6	1.79	0.87	1.36	1.94	68.74	**< 0.01**
q7	1.21	0.77	1.34	2.13	95.66	**< 0.01**
q8	1.75	0.73	1.50	2.37	23.03	0.81
q9	1.70	1.06	1.65	2.18	14.19	0.99
q10	1.83	0.78	1.51	2.28	14.61	0.99

(AIC = 22.157; BIC = 22.364; X^2^ = 318.9; *p* = 0.22)

Items were submitted to the analysis of invariance (DIF) according to sex, age group (< 30, 30–60 and > 60 years old) and type of disease (symptomatic or psychosocial). Several items disclosed non-uniform behavior according to sex, age and disease type (Table 5) after multivariate adjustment.

**Table 5:** Differential item functioning according to age group, sex and disease type (*n* = 1286)

Item	Sex	Age	Disease type
**q1**	F > M; *p* < 0.001	*p* = 0.492	SYM > P + S > PSY; *p* < 0.001
**q2**	F > M; *p* < 0.001	Y > A > O; *p* < 0.001	P + S > PSY; *p* < 0.001
**q3**	F > M; *p* < 0.001	Y > A > O; *p* < 0.001	P + S > PSY; *p* < 0.001
**q4**	F > M; *p* < 0.001	Y > O; *p* < 0.001	P + S > PSY > SYM; *p* < 0.001
**q5**	F > M; *p* < 0.001	Y > A > O; *p* < 0.001	P + S > PSY; *p* < 0.001
**q6**	F > M; *p* = 0.003	Y > A > O; *p* < 0.001	P + S > PSY > SYM; *p* = 0.044
**q7**	*p* = 0.055	A > Y > O; *p* = 0.004	P + S > PSY > SYM; *p* < 0.001
**q8**	F > M; *p* < 0.001	Y > A > O; *p* = 0.048	P + S > SYM > PSY; *p* < 0.001
**q9**	*p* = 0.300	Y > A > O; *p* < 0.001	P + S > SYM > PSY; *p* < 0.001
**q10**	F > M; *p* < 0.001	Y > A > O; *p* = 0.012	P + S > PSY; *p* < 0.001
**DLQI**	F > M; *p* < 0.001	Y > A > O; *p* = 0.004	P + S > PSY; *p* < 0.001

*F* female, *M* male, *O* age > 60 years, *A* age between 30 and 60 years, *Y* = age < 30 years; *PSY* psychological / social domain, *SYM* physical / symptomatic domain; *P + S* PSY + SYM

Post hoc test: Sidak sequential method

## Discussion

The DLQI proved to be a suitable instrument with which to evaluate HRQOL in a Brazilian population; however, there were psychometric concerns regarding the DLQI’s validity for different patterns of disease and its item composition.

The dermatological diseases studied in this work are among the most prevalent in Brazil, according to a recent survey [55]. The DLQI produced a wide variation in HRQOL scores, with a predominance of mild and moderate HRQOL impairment (49% of scores were between 2 and 10). This is characteristic of general dermatological diseases that have little impact on HRQOL [11, 13, 16, 23–26, 29–33, 56–60]. As such, highly discriminative instruments are required in this range of mild diseases. To date, this is the largest South American study on DLQI psychometrics.

The diseases with the highest DLQI score were urticaria, bullous dermatoses and hidradenitis. These are diseases whose physical symptoms are highly evident, demonstrating that the DLQI adequately assesses concrete discomforts [1]. Asymptomatic diseases, such as vitiligo, alopecia and melasma, can manifest as a different structural pattern of DLQI items. Some authors have found that the DLQI is unable to adequately measure the impact of mild diseases on patients’ HRQOL, which may, for example, present as lower responsiveness [16, 31]. In our study, five items presented a “ground” effect, which may have exacerbated this problem.

In 8 of the 10 questions, response options include “not at all” and “not relevant,” both of which are graded zero. Though these options contribute identically to the final score, they represent different meanings. Moreover, some items, such as q6 (sports) and q9 (sexual life), may elicit different response patterns based on the questioning period, since the instrument evaluates only the last 7 days [13, 16, 24, 61]. A recent proposal to adjust the total DLQI score of the questionnaire for the number of “not relevant” responses (DLQI-R) has the potential to improve the discriminatory power of the instrument, though a systematic psychometric study on item performance has not been performed [61, 62].

The correlation between item grades and the total score was adequate, except for item q9 (sexual life). Previous studies have found this item problematic [20, 61]. In a Chinese study that evaluated the DLQI among patients with neurodermatitis, q9 did not fit the model. The authors hypothesized that such a problem could be explained by the cultural difficulty of discussing this matter within the population [20]. Another possible explanation for this item’s performance is that the survey was conducted among adolescents or the elderly, for whom sexual activity is not a highly present element in daily life [61]. Moreover, IRT analysis indicates that q9 is most affected with severe HRQOL impact.

Due to the inter-item correlation, q9 must be carefully evaluated in different populations, since its exclusion from the questionnaire would make comparisons with other studies unfeasible. A practical proposal to address this would be the inclusion of a pre-assessment of the relevance of sexual activity in the participants’ lives, allowing the separation of groups on this issue, as occurs with item q7, related to work and study activities. Another proposal is the adaptation of the questionnaire scores (DLQI-R) that resizes the weight of all items marked “not relevant” [61, 62].

The current literature questions the dimensionality of the instrument [13, 18]. Our results evidence unidimensionality using the Horn parallel analysis and additional methods, which confirm the dimensionality of the original structure, as proposed by the author [5, 35–37]. The proper use of exploratory factor analysis for ordinal data (using polychoric correlations), rather than continuous data, can justify this previous divergence in comparison to some more robust methods [63].

The IRT analysis more accurately evaluated the HRQOL assessment questionnaires than the classical test theory psychometrics. The analysis of the DLQI by Rasch models among patients with psoriasis, atopic dermatitis and neurodermatitis revealed the lack of adjustability of some items, even though studies on the classical theory of the tests have indicated their adequacy [16, 20]. The Rasch-based analysis for ordinal data does not consider the independence of the discrimination (a-parameter) for each item, reducing the flexibility of the model.

In our study, Samejima’s graded response model produced the best performance in the IRT analysis and evidenced insufficient adjustment for items q6 (sports) and q7 (working/studying). The other studies that used Rasch models did not describe them in detail, from which it can be inferred that the differences in the fit of the items may be due to the underlying model [13, 16, 19, 20]. Additionally, when our data were analyzed according to other poorly fitting models (e.g., RSM and GRSM), the ordination of all items was not verified (data not shown). The practice of sports (q6) and work (q7) among the patients – especially those who were older and retired – was less likely to affect HRQOL. These particularities of different dermatosis and different population groups should be considered when using the DLQI for the assessment of HRQOL in specific circumstances.

Items q2 (embarrassment), q3 (shopping/gardening), q4 (clothing) and q5 (social/leisure activities) presented the best performance, suggesting that they address the fundamental elements of the different dermatoses studied. These items refer to activities and situations in which, in most cases, there is a certain degree of skin exposure, which could explain the discomfort experienced by patients with skin diseases. In this sample, symptoms (q1) and feelings (q2) were the first HRQOL aspects affected by skin diseases, while sexual life (q9) was the last.

The presence of DIF is a recurrent finding in recent literature on the DLQI. In a study that evaluated patients with psoriasis and atopic dermatitis, DIF was found for age, gender and type of disease [16]. In this study, several items exhibited DIF, which suggests that the DLQI does not adequately assess HRQOL in individuals with specific characteristics. Moreover, some characteristics that affect HRQOL are directly associated with groups of patients and diseases, justifying the behavior of DIF when analyzed as a single disorder. These elements indicate that caution should be exercised in the comparative evaluation of HRQOL in different population subgroups (e.g., men vs. women) and diseases with different characteristics (e.g., with predominantly physical complaints vs. psychological impact) [64].

The DIF verified between the elderly and the youth could also be explained by the difference in the range of diseases that affect each age group or the type of physical activity performed. In a study on hand eczema, DIF was found for age in q7 (influence of dermatosis at work), which could be explained by the older population being retired [19]. In this study, DIF was also evidenced for gender in q5 (influence in sports practice), which may demonstrate male predominance in sports practice in that population [19].

A Chinese study on neurodermatitis found that people who are younger than 35 years experienced less impact on HRQOL than those over 35 [20]. In another Chinese study with 9845 patients, Rasch’s analysis showed DIF for disease (q1, q2 and q5) and geographic location (q7, work/study), suggesting that the comparison between patients with different diseases can be limited [13].

Another study comparing the performance of the DLQI between different populations revealed DIF among cultures, despite using a questionnaire validated for the local language. Disease was found to present differently in interpretations of HRQOL among different cultures [17].

The ordering of the responses (grades within items) has also been questioned by other investigators. This may influence clinical decisions because some therapeutic protocols adopt the DLQI score, for which linearity is fundamental. Our results indicate a high correlation between the items and the total score. Furthermore, the ordering was adequate by IRT analysis, probably due to the use of Samejima’s model that disclosed greater adjustment to the data, which had not been used by previous studies (e.g., the Rasch-based model).

As the main contribution, this study confirms the DLQI is suitable for use in the Brazilian population. However, despite its unidimensionality, most items revealed non-uniform behavior according to sex, age and type of disease. Cautious interpretation is required to interpret a final score, especially if assessing or comparing heterogeneous samples. Under those conditions, the difference in HRQOL measures can be influenced by population characteristics rather than the disease burden. In practice, the best performance of the DLQI is achieved in assessing one kind of disease in samples with low variability in age and, preferably, of the same sex.

The strength of this study relies on the substantial sample and the diversity of dermatological diseases assessed, representing the most prevalent dermatoses among the Brazilian population [55]. Limitations are related to the unknown percentage of incomplete data or refusal to participate in the original studies, as well as which and how many items were marked “not at all” and “not relevant,” as it was a reanalysis of pooled data.

Further psychometric comparison of the DLQI with other multidimensional generic dermatological HRQOL questionnaires (e.g., Skindex-16), as well as testing of the DLQI’s responsiveness, temporal stability and exploration of its network structure (network analysis), are needed. Furthermore, the importance of the “not relevant” and “not at all” grades to the DLQI’s overall performance should be further assessed.

## Conclusions

The DLQI exhibits adequate reliability and a unidimensional structure for assessing HRQOL in Brazilian dermatological patients. Item performance varies according to sex, age and type of dermatosis, suggesting that these factors can result in different indications in the HRQOL assessment of patients. Researchers should be aware of these points when using the DLQI for evaluating HRQOL. Therefore, it is important to consider not only the numerical result of the DLQI in clinical decisions but also or the context of the patient’s responses that may affect the final score.

## Data Availability

The datasets used and/or analysed during the current study are available from the corresponding author on reasonable request.

## Abbreviations

AIC: Akaike information criterion
BIC: Bayesian information criterion
CFI: Comparative fit index
DIF: Differential item functioning
DLQI: Dermatology Life Quality Index
DLQI-BRA: DLQI for the Portuguese language in Brazil
ECV: Explained Common Variance
GPCM: Generalized partial credit model
GRSM: Graded ratings scale model
HRQOL: Health-related quality of life
IRT: Item response theory
MIREAL: Mean of Item Residual Absolute Loadings
RMSEA: Root mean square error of approximation
RSM: Rasch rating scale model
UniCo: Unidimensional Congruence
